# Fighting Tomato Fungal Diseases with a Biocontrol Product Based on Amoeba Lysate

**DOI:** 10.3390/plants12203603

**Published:** 2023-10-18

**Authors:** Sandrine Troussieux, Annabelle Gilgen, Jean-Luc Souche

**Affiliations:** R&D Department, Amoéba, 69680 Chassieu, France

**Keywords:** *Phytophtora infestans*, *Oidium neolycopersici*, *Leveillula taurica*, biofungicide, *Willaertia magna* C2c Maky

## Abstract

New solutions to reduce the use of chemical pesticides to combat plant diseases and to meet societal and political demands are needed to achieve sustainable agriculture. Tomato production, both in greenhouses and in open fields, is affected by numerous pathogens. The aim of this study is to assess the possibility of controlling both late blight and powdery mildew in tomatoes with a single biocontrol product currently under registration. The biocontrol product AXP12, based on the lysate of *Willaertia magna* C2c Maky, has already proved its efficacy against downy mildew of grapevine and potato late blight. Its ability to elicit tomato defenses and its efficacy in the greenhouse and in the field were tested. This study establishes that AXP12 stimulates the tomato genes involved in plant defense pathways and has the capacity to combat in greenhouse and field both late blight (*Phytophtora infestans*) and powdery mildew (*Oidium neolycopersici* and *Leveillula taurica*) of tomato.

## 1. Introduction

The amoeba *Willaertia magna* C2c Maky was isolated in 1998 from thermal waters in Aix-les-Bains (France). This free-living amoeba has demonstrated strong anti-microbial properties in its living form [1,2] as well as in its dead form as a lysate [3,4]. The lysate of *W. magna* C2c Maky is able to stimulate plant defense genes in grapevine and potato and possesses the ability to inhibit the release and germination of *Plasmopara viticola* and *Phytophtora infestans* [3,4]. These properties are exploited by a French biotech company, Amoéba (https://amoeba-nature.com/en/ (accessed on 11 October 2023)), for the development of plant protection products that can reduce the use of, or even replace, chemical pesticides. This is in accordance with European Union policy [5]. On the one hand, stable agricultural production still depends on the widespread use of chemical pesticides, while on the other hand, pesticide use is associated with negative impacts on the environment and human health [6,7]. A reduction in their usage and the replacement of specific active substances, as identified in Regulation (EC) No 1107/2009, the so-called ‘candidates for substitution’, is of very high importance for policymakers. At the same time, the agrifood sector’s primary concern is how to maintain high yields in a changing environment with increasing and re-occurring pathogen and pest pressures. To replace these active substances, alternative strategies, new active substances, and innovative solutions are urgently needed. This is especially challenging for row crops with high pathogen pressure, such as tomatoes.

Tomato was first imported to Europe in the 16th century and is nowadays an economically important crop traded in the fresh market and in the processing industry [8]. The estimated total world production of tomatoes in 2021 was 189 million tons; China was the largest producer, accounting for 36% of worldwide production [9]. Tomato production in 2021 in the European Union (EU) totaled 24 million tons [9]. One of the most destructive diseases is the late blight caused by *P. infestans*, which can affect stems, leaves, and fruit and can lead to a total crop loss within only two weeks. This disease re-emerged in North America in the 1980s–1990s [10]. Control of the disease can be achieved using phenylamide fungicides such as mefenoxam; however, biological solutions are preferred to avoid damage to the environment and health [11,12,13]. Moreover, *P. infestans* increasingly evade control efforts thanks to a stealthy lifetime helping to evade plant defenses and thanks to an adaptative genome with many transposable elements favoring genetic evolution [14,15]. Bordeaux mixture, based on copper, is often used to fight tomato late blight. The European Commission considers that copper compounds are candidates for substitution as they are persistent (the half-life in soil is greater than 120 days) and toxic (the long-term no-observed effect concentration for aquatic organisms is less than 0.01 mg/L) substances. Hence, by the end of 2018, the European Commission considered it appropriate to restrict the use of plant protection products containing copper compounds to a maximum application rate of 28 kg/ha of copper over a period of 7 years (i.e., on average 4 kg/ha/year) in order to minimize the potential accumulation in soil and the exposure for not target organism [16]. As a consequence, biological alternatives are in high demand.

Powdery mildew is another disease that can affect tomatoes, and it can be caused by two kinds of microorganisms: *Oidium neolycopersici*, which causes severe powdery mildew on all aerial parts of the tomato, excluding the fruit [17,18,19,20], and *Leveillula taurica*, an obligate fungal pathogen that causes endoparasitic powdery mildew disease on a broad range of plants, including tomato [21]. Powdery mildew can be managed and controlled by active ingredients such as benomyl, bitertanol, bupimirate, and carbendazim, but also by bioassimilable sulfur [22].

The objective of this study is to evaluate if the lysate of *W. magna* C2c Maky is able to control several tomato pathogens and could, therefore, help to reduce or replace the use of chemical pesticides in greenhouse and field conditions.

## 2. Results

### 2.1. Elicitor Effect on Tomato Genes

The purpose of this approach is to assess the elicitor activity of one formulated product (AXP12) and of the pure active substance (AXP10) on whole plants of the tomato variety Money Maker. The experiment sought to compare the level of activation of salicylic acid (SA)-dependent plant defense pathways in response to these two products. Tomatoes were grown in pots for 3 weeks and were treated by being sprayed with AXP10 at two different rates or with AXP12 at a single rate. Plants were harvested 24 h after treatment, and the level of defense pathway activation was determined by RT-qPCR.

**Figure 1:**
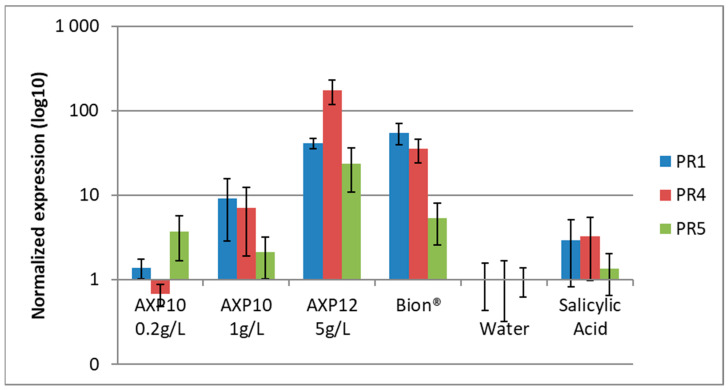
Normalized expression of the PR1, PR4, and PR5 genes in tomato plants. The results are expressed as the mean +/− standard deviation (n = 8, three independent replicates); AXP10 is the raw active substance tested at two concentrations; AXP12 is the formulated product tested at 5 g/L (containing 1 g/L of active substance). Bion^®^ was used at 0.015% as a positive control for elicitation, water was used as negative control and was used to normalize the level of expression of the marker genes, and salicylic acid at 1 mM was used as hormonal positive control.

The treatment with SA (hormonal positive control) slightly activates the three marker genes by 3×, 3.2×, and 1.3× for PR1, PR4, and PR5 genes, respectively (Figure 1).

The Bion^®^ (Syngenta, Bâle, Switzerland) treatment (elicitor positive control), containing 50% of acibenzolar-S-methyl, strongly activates these three marker genes by 55×, 35.1×, and 5.3× for PR1, PR4, and PR5 genes, respectively (Figure 1).

APX10 at 0.2 g/L does not induce the activation of the PR1 and PR4 genes and slightly (3.7×) activates the PR5 gene, whereas APX10 at 1 g/L activates these three genes by 9.2×, 7.1×, and 2.1× for the PR1, PR4, and PR5 genes, respectively (Figure 1).

The formulated product, AXP12, at a dose of 5 g/L containing 1 g/L of active substance, induces very strong activation of the three genes by 41.2×, 173.2×, and 23.5× for the PR1, PR4, and PR5 genes, respectively (Figure 1).

The results showed the activation of the SA-dependent defense pathway specifically in response to the presence of the active substance with a dose-response effect and of the formulated product. The three marker genes were clearly activated following the application of APX10 at 1 g/L and APX12 at 5 g/L (ΔΔ-SQ > 2), compared to activation resulting from the water treatment. This activation is also higher than the activation induced by the SA treatment (hormonal positive control). Moreover, the level of activation of these three genes was higher in the plants treated with the formulated product (APX12 at 5 g/L containing 1 g/L of active substance) than in plants treated with the active substance alone (APX10) at 1 g/L.

Indeed, the formulated product induced a response almost six times stronger than the active substance regarding the expression of PR1, a response almost five times stronger regarding the expression of PR4, and a response 2.5 times stronger regarding the PR5 gene. Therefore, the formulation of the APX12 product may improve the activity of the active ingredient APX10 for better plant protection.

### 2.2. Efficacy against Diseases

#### 2.2.1. Tomato Late Blight

**Figure 2:**
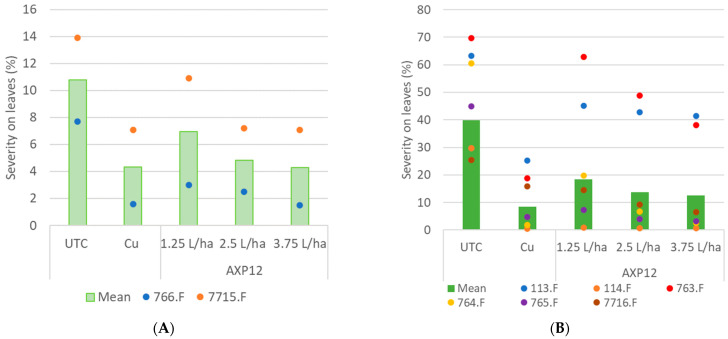
Tomato late blight severity on leaves: (**A**), under mild infestation; (**B**), with medium to high infestation. Bars represent the means of all trials within the same condition; dots represent the severity in each trial. UTC: Untreated control; Cu: copper treatment at 2 kg/ha; AXP12 was tested at three doses: 1.25, 2.5, and 3.75 L/ha.

The fight against *P. phytophtora*, responsible for tomato late blight, was studied in six field trials and two greenhouse experiments conducted in 2022. The level of disease in the untreated plants varied from 7.7% in the 766.F trial to 69.7% in the 763.F trial. To better observe the results, data were split to represent cases where the occurrence of disease was lower than 20% (Figure 2A) and trials where the occurrence of disease was higher than 20% (Figure 2B). In the case of low infestation (766.F and 7715.F trials), AXP12 at 3.75 L/ha was statistically as efficient as copper, with 81% and 49% efficacy in the 766.F and 7715.F trials, respectively (Figure 2A, Table 1). Three trials were highly infected (above 60% infestation), and the efficacy of copper dropped to 60% and 74% in the 113.F and 763.F trials, respectively. AXP12 efficacy dropped to 33% and 45% in these two trials, respectively. However, the efficacy was maintained in the 764.F trial, even though the disease reached 61% in the untreated plots, with 97% efficacy for the highest dose of AXP12, which was statistically as efficient as copper (Figure 2B, Table 1). Increasing the AXP12 dose resulted in increased efficacy in all cases (Figure 2).

**Figure 3:**
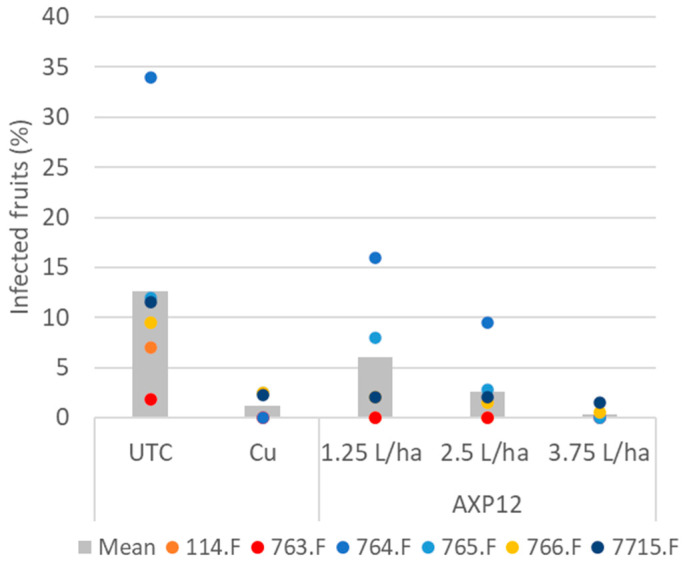
Percentage of fruits infected with *P. infestans*. Bars represent the means of all trials within the same condition; dots represent the severity of each trial. UTC: Untreated control; Cu: copper treatment at 2 kg/ha; AXP12 was tested at three doses: 1.25, 2.5, and 3.75 L/ha.

Fruits were contaminated by *P. infestans* in six trials (114.F, 763.F, 764.F, 765.F, 766.F, and 7715.F) at up to 34% in trial 764.F. No disease was observed with copper treatment in this trial. A dose effect was observed in tomatoes treated with AXP12, with no disease on fruit at the highest dose, which was statistically as efficient as copper (Figure 3, Table 2). In the other trials, the mean disease severity ranged from 1.8% to 12%. All treatments were significantly different from the untreated plots and similar to copper, at least for the highest dose of AXP12 (Figure 3, Table 2). In the 765.F, 766.F, and 7715.F trials, the highest dose of AXP12 tended to be more efficient than copper, but this effect was not statistically significant (Figure 3, Table 2).

#### 2.2.2. Powdery Mildew

**Figure 4:**
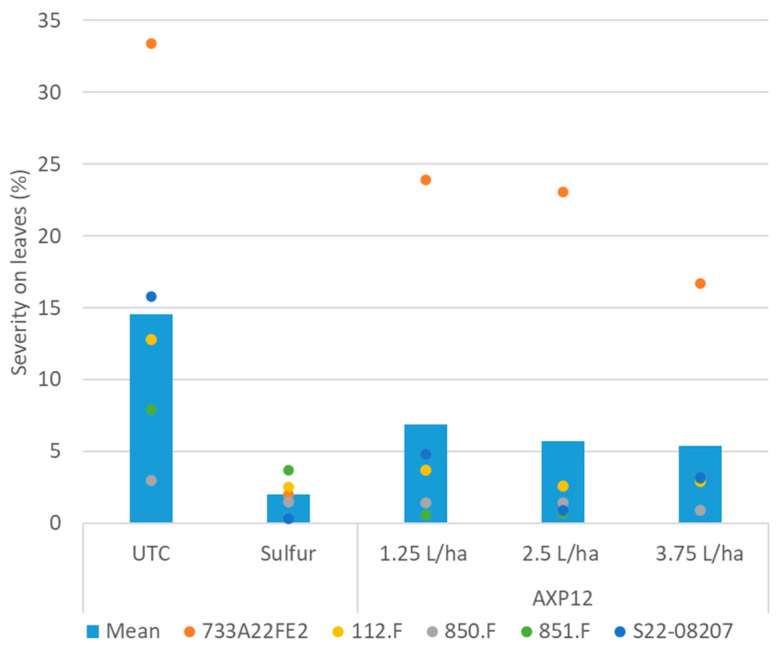
Powdery mildew severity on leaves. Bars represent the means of all trials within the same condition; dots represent the severity of each trial. UTC: Untreated control, sulfur treatment was applied at 5 kg/ha; AXP12 was tested at three doses: 1.25, 2.5, and 3.75 L/ha.

Powdery mildew on tomato was caused by *O. neolycopersi* (733A22FE2, S22-08207, 850.F, and 851.F trials) and *L. taurica* (112.F and S22-08207 trials). The attack severity was above 20% only in the 733A22FE2 trial, where it reached 33%. Sulfur treatment was able to contain *O. neolycopersi* attack with 94% efficacy, whereas AXP12 protection at the higher rate was limited to 50% efficacy. However, in all other cases, AXP12 was statistically as efficient as sulfur when the disease severity was between 10 and 20% in the untreated plots and was statistically more efficient than sulfur in the 851.F trial at the lowest and medium doses in the case of low infestation (8%) (Figure 4, Table 3).

## 3. Discussion

Worldwide, tomato production is harmed by many fungal diseases, such as buckeye rot and corky roots caused by several oomycete pathogens, *Fusarium* wilt, crown and root rot, and *Verticilium* wilt [23]. Late blight of tomato caused by *P. infestans* re-emerged in the late 1980s [10], as well as powdery mildew [20], particularly in greenhouses where there is economic pressure for lower temperatures to reduce production costs resulting in a recrudescence of powdery mildew [17]. In this study, we evaluated the possibility of fighting late blight and powdery mildew of tomato with a single biocontrol product based on the lysate of a free-living amoeba, *W. magna* C2c Maky. As this product was demonstrated to have a dual mode of action in grapes and potatoes, namely a direct anti-germinative effect and an indirect action by stimulating plant defenses [3,4], both properties were evaluated in tomatoes.

After considering previous data on plant defense elicitation [3,4], pathogenesis-related (PR) genes were targeted. The purpose of this approach was to assess the ability of the formulated product AXP12 and of two rates of the raw active substance AXP10 to elicit plant defenses by comparing the level of activation of the salicylic acid (SA) plant defense pathway using reverse transcription-quantitative polymerase chain reaction (RT-qPCR). The role of SA has been demonstrated in tomato in induced resistance against powdery mildew [24]. The consequence is a reduction in damaged tissues in the presence of the pathogenic strain due, in part, to the induction of the PR genes [25]. When a pathogen is recognized by the plant, it triggers a signaling network, activating defense genes encoding, for example, PR proteins that degrade the pathogenic cells [26]. The effect of AXP10 and AXP12 on PR gene activation was compared to that of Bion^®^, which contains 2.35 mM benzotiadiazol, a derivative of SA known to induce resistance in tomato [27,28], and 1 mM SA. The activation of PR1 and PR4 genes by SA was low (3×), and the PR5 gene was not activated, whereas Bion^®^ increased the expression of the PR1, PR4, and PR5 genes by a factor of 55, 35, and 5, respectively. AXP10 at 1 g/L induced increases of nine-fold in PR1 and seven-fold in PR4, with a dose-dependent effect as no activation was observed with the diluted dose of 0.2 g/L. The formulated product AXP12 greatly improved the elicitation property, causing a 41-, 173-, and 24-fold increase in the expression of the PR1, PR4, and PR5 genes, respectively. The presence of wetting agents in the formulation may be responsible for activation of the plant defenses by increasing the spreading and coverage of the product on plant leaves. The PR1 protein is known to enhance tomato defenses and to be involved in the resistance of tomato to *P. infestans* [29,30] and to reduce sporangia germination and germ-tube length [31]. Some PR proteins have enzymatic activities; for example, β-glucanases (PR-2) are involved in hydrolytic activities [32], and PR5 proteins can cause the osmotic rupture of fungal membranes [26]. Other biocontrol substances, such as *Glycyrrhiza glabra* leaf extract, can provoke the induction of the PR1, PR2, and/or PR5 genes [33,34]. These genes are also naturally induced in the presence of a pathogenic strain such as *Xanthomonas campestris* [25]. There are, of course, many other ways to activate plant defenses for biocontrol products. For example, *Trichoderma harzanium* is able to boost the jasmonic acid pathway and induce a systemic defense response in planta [34].

In addition to this elicitor property, AXP12 was shown to inhibit spore germination of *Plasmopara viticola*, and its efficacy in the field to fight downy mildew of grape and potato late blight was demonstrated [3,4]. Tomato late blight is caused by the same pathogenic strain as potato late blight, *P. infestans*, and is responsible for important crop losses [12,35,36]. The best control is actually obtained by spraying chemical fungicides, such as mefenoxam, a phenylamide fungicide [37]. As more and more strains of *P. infestans* become resistant to chemical fungicides, alternative solutions are needed to maintain tomato yield. *Trichoderma harzanium*, *Bacillus* strains, oak bark compost, plant extracts, fungal endophytes, *Pseudomonas aeruginosa*, and *Lysobacter enzymogenes*, among others, demonstrate activity in vitro and in planta against tomato late blight [11,38,39,40,41,42,43,44], but their efficacy is not proven in field trials. Synthetic peptides such as NoPv1 are able to inhibit the biosynthesis of the appressorium, which is essential for the pathogenicity of *P. infestans* [45], but once again, the efficacy was only demonstrated in vitro. The precise mode of action is not always deciphered, as for the amoeba lysate or fungal *Chaetomium* extracts [46], mainly because the majority of molecules are unknown [47]. The efficacy of biocontrol products is also linked to the soil quality and its bacterial and fungal populations [11]. For example, *Pseudomonas aeruginosa* FG106 produces proteases, lipases, siderophores, ammonia, indole acetic acid, and hydrogen cyanide and forms biofilms that facilitate biocontrol of pathogens [40]. *Bacillus velezensis* KOF112 is an endophyte microorganism able to inhibit the mycelial growth of *P. infestans* [41]. These studies exemplify the diversity of actions that can be achieved through biocontrol procedures.

Powdery mildew of tomatoes is also involved in crop loss. This disease is well controlled in greenhouses by combining high temperatures and low humidity [17]; however, this solution has a high energy cost, and due to the increasing prices of electricity, producers are reducing their energy expenses, and the disease has returned with infection rates reaching 90% [19,48]. This disease can be reduced by the spread of sulfur, which has negligible toxicity to animals, insects, and plants [22]. The two powdery mildews have two different modes of contamination, which allows us to discriminate them. *O. neolycopersi* only develops on the upper leaf surface [17,18], while *L. taurica* develops inside the leaf and becomes visible under the leaf when it emerges from the stomata [21].

The biocontrol product AXP12 possesses very interesting properties as it is able to fight *P. phytophtora*, *O. neolycopersi*, and *L. taurica* with up to 97% efficacy on leaves and 100% efficacy on fruits under field and greenhouse conditions. In all conditions and trials, AXP12 was not phytotoxic to tomato leaves and fruits. After commercialization, expected in 2025 under the trade name AXPERA EVA, the product may be associated with integrated pest management strategies such as selecting resistant tomato varieties, rotating crops, and avoiding planting potatoes in the same area [13]. In the case of high infestation, the efficacy could be increased by using AXP12 in combination with reduced doses of copper, sulfur, or other fungicides.

To conclude, we have demonstrated that a single biocontrol product, AXP12, can be used to fight both late blight and powdery mildew of tomatoes. The next steps will include other targets, such as cladosporiosis and early blight on tomatoes, as well as other pathosystems.

## 4. Materials and Methods

### 4.1. Active Substance and Formulation

The active substance (AS) is the lysate of the amoeba W. magna C2c Maky, named AXP10, in its dry form [3]. Briefly, after cultivation in a bioreactor, the amoeba culture was centrifugated (2500× *g*, 15 min at room temperature) to remove the culture medium, then mechanically lysed and dried. This powder was formulated into an aqueous suspension concentrate, named AXP12, containing 20% AS (*w*/*w*).

### 4.2. Stimulation of Plant Defense PR Protein Genes

#### 4.2.1. Plant Material and Products

The tomato (*Solanum lycopersicum*) variety used for this study is Money Maker. In the study, 108 plants were grown in pots on fertilized soil for 3 weeks in a greenhouse (at 22 °C with 14 h light per day).

Two products were studied: AXP10 at 0.2 and 1 g/L and AXP12 at 5 g/L, which contained 1 g/L of AS.

#### 4.2.2. Plant Treatment and Sampling

Plantlets were sprayed three times for each condition every 24 h (Table 4). Bion^®^ was used at 0.015% according to the manufacturer’s instructions (Syngenta, Switzerland) as a positive control for elicitation [27]. Water was used as a negative control. Salicylic acid at 1 mM was used as hormonal positive control. Plantlets were harvested 24 h after the last treatment and immediately frozen in liquid nitrogen until RNA extraction. These treatments were conducted three times, independently.

#### 4.2.3. RT-qPCR Analysis

RNA was extracted with the EZNA Plant RNA Kit (Omega Bio-Tek, VWR International, Fontenay-sous-Bois, France) and retrotranscribed by the iScript cDNA synthesis kit (BIO-RAD, Roanne, France). The level of expression of three marker genes, reflecting the activation rate of the salicylic acid pathway (genes encoding PR1, PR4, and PR5 proteins), and of two control references (18S rRNA gene and *act*) was measured by qPCR with the iTaq Universal SYBR^®^ Green Supermix (BIO-RAD). The primers used are described in Table 5.

Therefore, five analyses were performed for each condition in triplicate. For each analysis, a Ct value was extracted and then normalized with standard curves in order to obtain an SQ value, which takes into account the efficiency of the primers. In all conditions, the levels of expression of marker genes were determined in comparison to the reference gene (the housekeeping gene). For this purpose, the SQ values obtained for the marker genes were divided by SQ values obtained for the reference genes (normalized SQ = ΔSQ).

In all conditions, normalized expressions of the marker genes (=ΔΔSQ) were then calculated considering the level of expression of those genes in the untreated condition (water). For this purpose, the normalized SQ values obtained for these marker genes were divided by normalized SQ values obtained for the untreated condition.

As experiments were performed in triplicate, three ΔΔSQ values were obtained for each condition, and each gene was analyzed. These three ΔΔSQ values were averaged to obtain the level of activation for each gene per condition.

### 4.3. Greenhouse and Field Trials

#### 4.3.1. Trials with Phytophthora Infestans

Six field trials (FT) and two greenhouse (GH) experiments conducted in 2022 according to good experimental practices and to the European and Mediterranean Plant Protection Organization guidelines are reported herein, with six trials located in Italy and two in Spain (Table 6).

Treatments were sprayed on leaves once a week for 6 weeks (ABCDEF). The formulation AXP12 was applied at three rates (1.25, 2.5, and 3.75 kg/ha) and compared to tribasic copper sulfate (TBCS) at one rate (2 kg/ha) (Table 7). The disease severity on 100 leaves and 50 fruits was assessed.

The efficacy and selectivity of each treatment were evaluated on the last assessment, seven days after the last treatment (F).

#### 4.3.2. Trials with *Oidium neolycopersici* or *Leveillula taurica*

Three trials were conducted against *O. neolycopersici* (733A22FE2 and 850.F trials on Pixel tomato variety, 851.F trial on DRW7723 tomato variety, and S22-08207 on clementine tomato variety) in Italy in 2022 and two trials were conducted against *L. taurica* (112.F trial on Maraskino tomato variety in Italy in 2022 and S22-08207 trial on the clementine variety in The Netherlands in 2022).

In the 733A22FE2 field trial, the efficacy and the selectivity of the product AXP12 at 1.25, 2.5, and 5 L/ha against powdery mildew (*O. neolycopersici*) were evaluated on 100 leaves after eight treatments (ABCDEFGH) in comparison to the reference Thiovit^®^ Jet Microbilles (80% sulfur, Syngenta) at 5 kg/ha.

In the 850.F and 851.F greenhouse trials, the efficacy and the selectivity of the product AXP12 at 1.25, 2.5, and 3.75 L/ha against powdery mildew (*O. neolycopersici*) were evaluated on 100 leaves after eight treatments (ABCDEFGH) in comparison to the reference Thiovit^®^ Jet Microbilles at 5 kg/ha.

In the 112.F greenhouse trial, the efficacy and the selectivity of the product AXP12 at 1.25, 2.5, and 5 L/ha against powdery mildew (*L. taurica*) were evaluated on 100 leaves after eight treatments (ABCDEFGH) in comparison to Thiovit^®^ Jet Microbilles at 5 kg/ha (8 treatments).

In the S22-08207 greenhouse trial, the efficacy and the selectivity of the product AXP12 at 1.25, 2.5, and 3.75 L/ha against both powdery mildew (*O. neolycopersici* and *L. taurica*) were evaluated on 100 leaves after seven treatments (ABCDEFG) in comparison to the reference Thiovit^®^ Jet Microbilles at 7.5 kg/ha.

#### 4.3.3. Statistical Analysis

Data from assessments were analyzed by variance analysis (ANOVA) with ARM 2022.5 software (Gylling Data Management, Brookings, SD, USA). If a significant effect of the treatment was obtained (on the basis of the ANOVA), differences between means were checked with the Student–Newman–Keuls (SNK) test (*p* = 0.05).

Statistical significance was indicated by a letter. Treatments marked with different letters were significantly different in accordance with the Student–Newman–Keuls (SNK) test [52,53] conducted at a 95% confidence level.

## Figures and Tables

**Table 1:** Statistical significance of treatments in tomato late blight on leaves.

Trial Number	UTC *	Copper 1.4 L/ha	1.25 L/ha	AXP12 2.5 L/ha	3.75 L/ha
113.F	a	c	b	b	b
114.F	a	b	b	b	b
763.F	a	c	a	b	b
764.F	a	d	b	c	d
765.F	a	b	b	b	b
766.F	a	b	b	b	b
7715.F	a	b	b	b	b
7716.F	a	b	b	c	d

* UTC: Untreated control; blue letters indicate equivalent efficacy between AXP12 and copper treatments; letters a, b, c, and d: the same characters indicate that data are not significantly different (*p* = 0.05), green letters indicate better efficacy of AXP12 compared to copper, blue letters indicate equivalent efficacy between AXP12 and copper treatments.

**Table 2:** Statistical significance of treatments in tomato late blight on fruit.

Trial Number	UTC *	Copper 1.4 L/ha	1.25 L/ha	AXP12 2.5 L/ha	3.75 L/ha
114.F	a	b	b	b	b
763.F	a	b	b	b	b
764.F	a	d	b	c	d
765.F	a	c	b	c	c
766.F	a	b	b	b	b
7715.F	a	b	b	b	b

* UTC: Untreated control; letters a, b, c, and d: the same characters indicate that data are not significantly different (*p* = 0.05); blue letters indicate equivalent efficacy between AXP12 and copper treatments.

**Table 3:** Statistical significance of treatments in tomato powdery mildew on leaves.

Trial Number	UTC *	Sulfur 7.5 kg/ha	1.25 L/ha	AXP12 2.5 L/ha	3.75 L/ha
733A22FE2	a	d	b	b	c
112.F	a	b	b	b	b
850.F	a	b	b	b	b
851.F	a	c	b	b	c
S22-08207.F	a	c	b	c	b

* UTC: Untreated control; letters a, b, c and d: the same characters indicate that data are not significantly different (*p* = 0.05); blue letters indicate equivalent efficacy between AXP12 and sulfur treatments; green letters indicate better efficacy of AXP12 compared to sulfur.

**Table 4:** Studied conditions.

Product	Concentration	Treatment
AXP10	0.2 g/L	First spraying
		Second spraying
		Third spraying
AXP10	1 g/L	First spraying
		Second spraying
		Third spraying
AXP12	5 g/L	First spraying
		Second spraying
		Third spraying
Water	/	First spraying
Water	/	Second spraying
Bion^®^	0.015%	Third spraying
Water	/	First spraying
		Second spraying
		Third spraying
Water	/	First spraying
Water	/ 1 mM	Second spraying
Salicylic acid		Third spraying

**Table 5:** Primers used.

Target Gene	Primer Name	Primer Sequence	Reference
18S-1	Forward	AAAAGGTCGACGCGGGCT	[49]
	Reverse	CGACAGAAGGGACGAGAC	
*act*	Forward	CACCACTGCTGAACGGGAA	[50]
	Reverse	GGAGCTGCTCCTGGCAGTTT	
*loxA*	Forward	TGAACCATGGTGGGCTGAAA	[50]
	Reverse	CTGCCCGAAATTGACTGCTG	
PR1-3	Forward	GCACTAAACCTAAAGAAAAATGGG	[50]
	Reverse	AAGTTGGCATCCCAAGACATA	
PR4-1	Forward	ATGGGGTTGTTCAACATCTCATTGTTACT	[51]
	Reverse	TTAATAAGGACGTTCTCCAACCCAGTT	
PR5-1	Forward	CCCCAACAAAACCTAGTGGA	[32]
	Reverse	ACCAGGGCAAGTAAATGTGC	

**Table 6:** Trial characteristics.

Trial Reference	Type	Country	Variety
113.F	Open Field	Italy	Fokker
114.F	Open Field	Italy	Nunhems 6438
763.F	Open Field	Italy	Fokker
764.F	Open Field	Italy	Heinz
765.F	Open Field	Italy	Heinz
766.F	Greenhouse	Italy	Sir Elyan
7715.F	Open Field	Spain	Encomienda
7716.F	Greenhouse	Spain	Huevo de Toro

**Table 7:** Applied protocol. CONC.: concentration, AS: active substance, UTC: untreated control, TBCS: tribasic copper sulfate.

Modality	Product	Form	CONC.	Rate (kg/ha)	AS	Rate (g/ha)
1	UTC					
2	TBCS	WG	40%	2.00	copper	800
6	AXP12	SC	20%	1.25	AXP10	250
7	AXP12	SC	20%	2.50	AXP10	500
8	AXP12	SC	20%	3.75	AXP10	750

## Data Availability

Study reports (RT-qPCR, field trials) are available on request from the corresponding author.
